# Wound of the Heart Temporarily Healed

**Published:** 1844-12-28

**Authors:** 


### WOUND OF THE HEART TEMPORARILY HEALED.

BY M. MARINI.

A woman, 32 years of age, was stabbed with a dagger in the region of the heart, immediately fell, and lost a large quantity of blood. M. Marini, who saw her very shortly afterwards, found her just in life, covered with a cold clammy sweat, continually fainting, and with a wavering weak pulse. The wound was near the mamma, about two inches and a few lines from the edge of the sternum, between the fourth and fifth ribs. It was judged that the pericardium at least was injured, and that the wound was mortal. She was, therefore, carried to the hospital, and nothing was done till next day, when, as she was still alive, she was more carefully examined, and as the surgeons agreed that the wound was simply superficial and injured no important part, little was done, excepting drawing blood from the arm, or applying a few leeches to keep down threatened inflammation. Several times, however, she was in imminent danger of death. The external wound cicatrized, and she was dismissed as perfectly cured, after having been in the hospital six weeks. Three weeks after this, when rising suddenly from her bed one morning, she fell down and instantly expired.

The external cicatrix was found solid and perfect. The condensed tissue, which filled the line of the original wound, could easily be traced between the fourth and fifth ribs into the interior of the chest. Half-a-pound of colourless serous fluid was found in the left pleura, and the upper lobe of the lung had contracted many firm recent adhesions. The part of the pericardium where it had been penetrated by the dagger was much thickened, and presented traces of acute inflammation. A large cyst, of a blackish-blue colour, filled with partly fluid, partly coagulated blood, adhered by a large pedicle to the left side of the pericardium. The pericardium was filled with about two pounds of blood, partly fluid, partly clotted; the heart was atrophied, its coats thinned, and its cavities full of blood. Near its apex, it was pierced with a rounded conical-shaped aperture, which communicated with the left ventricle. The aperture, which was so large as to permit the introduction of an ordinary pair of forceps, was surrounded by a ring of soft whitish lymph, which appeared to have adhered to the pericardium at the point penetrated by the dagger, and to have prevented the further effusion of blood.—*Ed. Med. and Surg. Jour., from Il Raccog. Medico.*

